# Observation of a Flat and Extended Surface State in a Topological Semimetal

**DOI:** 10.3390/ma15082744

**Published:** 2022-04-08

**Authors:** Ryo Mori, Kefeng Wang, Takahiro Morimoto, Samuel Ciocys, Jonathan D. Denlinger, Johnpierre Paglione, Alessandra Lanzara

**Affiliations:** 1 Materials Sciences Division, Lawrence Berkeley National Laboratory, Berkeley, CA 94720, USA; ryomori@berkeley.edu (R.M.); sciocys@berkeley.edu (S.C.); 2 Applied Science & Technology, University of California, Berkeley, CA 94720, USA; 3 Maryland Quantum Materials Center, Department of Physics, University of Maryland, College Park, MD 20742, USA; kw624@physics.rutgers.edu (K.W.); paglione@umd.edu (J.P.); 4 Department of Applied Physics, The University of Tokyo, Hongo, Tokyo 113-8656, Japan; morimoto@ap.t.u-tokyo.ac.jp; 5 JST, PRESTO, Kawaguchi 332-0012, Japan; 6 Department of Physics, University of California, Berkeley, CA 94720, USA; 7 Advanced Light Source, Lawrence Berkeley National Laboratory, Berkeley, CA 94720, USA; jddenlinger@lbl.gov

**Keywords:** topological materials, photoemission spectroscopy, surface states, flat bands

## Abstract

A flat band structure in momentum space is considered key for the realization of novel phenomena. A topological flat band, also known as a drumhead state, is an ideal platform to drive new exotic topological quantum phases. Using angle-resolved photoemission spectroscopy experiments, we reveal the emergence of a highly localized surface state in a topological semimetal BaAl${}_{4}$ and provide its full energy and momentum space topology. We find that the observed surface state is localized in momentum, inside a square-shaped bulk Dirac nodal loop, and in energy, leading to a flat band and a peak in the density of state. These results imply this class of materials as an experimental realization of drumhead surface states and provide an important reference for future studies of the fundamental physics of correlated quantum effects in topological materials.

## 1. Introduction

Because of strong localization, flat band electronic states are considered to play a critical role in achieving strong electron correlations, leading to exotic quantum states of matter, such as high-$T_{c}$ superconductivity, magnetism, and fractional Hall effect. Such unique conditions are achieved in various systems, including Kagome lattices [1,2,3], twisted bilayer graphene [4], and topological materials [5,6,7,8,9,10,11]. In topological materials, one notable class of the topological non-trivial states—nodal line semimetals—are the neighbor states to various topological quantum phases, such as three-dimensional Dirac semimetals, Weyl semimetals, topological insulators, and spinful Weyl nodal line semimetals, and hence it is regarded as an ideal platform to study and control the quantum topological phase transition by breaking symmetries [12]. In a topological nodal line semimetal, the linearly degenerate bands cross each other on a mirror plane, giving rise to a nodal line in the momentum space. The crossing bands on the mirror plane cannot hybridize since they have opposite mirror eigenvalues, resulting in a stable nodal loop/line on the mirror plane. The projection of these bulk nodal lines onto the surface fills the inside of the nodal lines and generates the so-called drumhead surface state [5,6,7,8,9,10,11], given its resemblance to the head of an open drum. The peculiar momentum space structure of these states, flat and localized in energy, is critical for the realization of novel phenomena [13,14,15,16,17,18,19,20,21,22,23,24,25,26,27,28], such as topological superconductivity [14,16,17,18,29] and magnetism [1,2,3,15]. In the case of superconductivity, for example, the momentum extension of the drumhead state (area) is proportional to the pairing strength, and hence $T_{c}$ [14,29]. Therefore, topological semimetals exhibiting drumhead surface states present a significant expansion of topological materials beyond topological insulators and nodal-point Dirac/Weyl semimetals.

**Figure 1:**
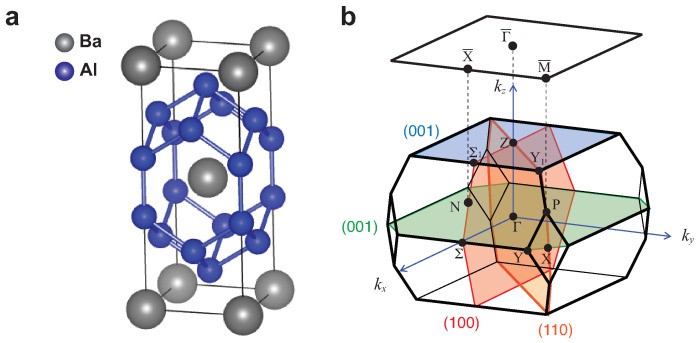
Crystal structure of the topological semimetal BaAl${}_{4}$. (**a**) The crystal structure of BaAl${}_{4}$. The gray and the blue spheres represent the Ba and the Al atoms, respectively. (**b**) The bulk Brillouin zone (BZ) and the (001) surface BZ, marked with high-symmetry points. In the bulk structure, the three non-equivalent mirror-reflection planes $m_{001}$ (green plane ($k_{z}=0$) and blue plane ($k_{z}=2\pi /c$)), $m_{110}$ (orange plane) and $m_{100}$ (red plane) are illustrated.

Recently, BaAl${}_{4}$ has been reported to have topological semimetallic features with 3D Dirac dispersions and possible nodal lines protected by crystal symmetry [30]. While this can explain several of the observed transport properties, such as extremely large magnetoresistance, including quantum oscillations [30], its associated surface-localized band is still elusive. In this paper, we use angle-resolved photoemission spectroscopy (ARPES), experimentally combined with theoretical calculation, to study in detail the electronic structure of BaAl${}_{4}$, focusing on the nodal line and the surface-localized bands. BaAl${}_{4}$ has the body-centered tetragonal structure in the space group of I4/mmm (No. 139), as shown in Figure 1a, also known as a prototype parent crystal of a large family of compounds [30,31,32,33,34]. The bulk and (001) surface-projected BZs with high-symmetry points labeled are shown in Figure 1b. The crystal has three non-equivalent mirror-reflection planes $m_{001}$ (green and blue planes), $m_{110}$ (orange plane), and $m_{100}$ (red plane) (see Figure 1b). The $m_{110}$ plane and $m_{100}$ plane have equivalent mirror planes along the orthogonal directions. Therefore, a number of Dirac nodal lines can exist on these planes when spin–orbit coupling (SOC) is negligible, leading to the presence of drumhead surface states.

## 2. Materials and Methods

Single crystals of BaAl${}_{4}$ were synthesized by a high-temperature self-flux method and characterized by X-ray diffraction at room temperature with Cu K${}_{\alpha }$ (λ=0.15418 nm) radiation in a powder diffractometer [30].

Electronic structure calculations were performed within the framework of the density functional theory (DFT) with the PAW pseudopotentials, as implemented in the Quantum Espresso package [35]. The generalized gradient approximation (GGA) with the Perder–Burke–Ernzerhof parameterization (PBE) was used [36]. A plane wave energy cut-off 40 Ry and 24 × 24 × 24 *k*-mesh to sample the BZ were used for the bulk calculations. Total energies were converged to smaller than $10^{-10}$. The experimental crystal data (a=b=4.566 Å, c=11.278 Å) were used [37]. The calculation of surface electronic structures was carried out with momentum-resolved local density of states of a semi-infinite surface by employing a tight-binding (TB) model obtained by using the Wannier90 [38] and WannierTools suite of code [39]. The quality of the Wannier function-based TB model was checked by comparing it with the DFT calculation (see Figure S3 in Supplementary Materials (SM)). The comparison of our experimental spectra with the theory-calculated surface states shows good agreement with a 25% expansion in the energy dimension of the calculation result. The VESTA package was used for visualization of the crystal structure [40].

ARPES measurements on single crystalline samples of BaAl${}_{4}$ were performed at the Beamline 4.0.3. end station of the Advanced Light Source in Berkley, CA, USA. Samples were cleaved in situ to yield clean (001) surfaces and measured at 20 K in an ultra-high vacuum better than $3\times 10^{-11}$ Torr, using the photon energy of 80–128 eV with a Scienta R8000 analyzer. The energy resolution was 20–30 meV and the angular resolution was better than $0.2^{\circ }$ for all measurements. According to these photon energy dependence measurements, the inner potential of BaAl${}_{4}$ is estimated at 10.5 eV. Partial ARPES data in this paper were analyzed using pyARPES, an open-source python-based analysis framework [41].

## 3. Results

### 3.1. Bulk Dirac Nodal Line

**Figure 2:**
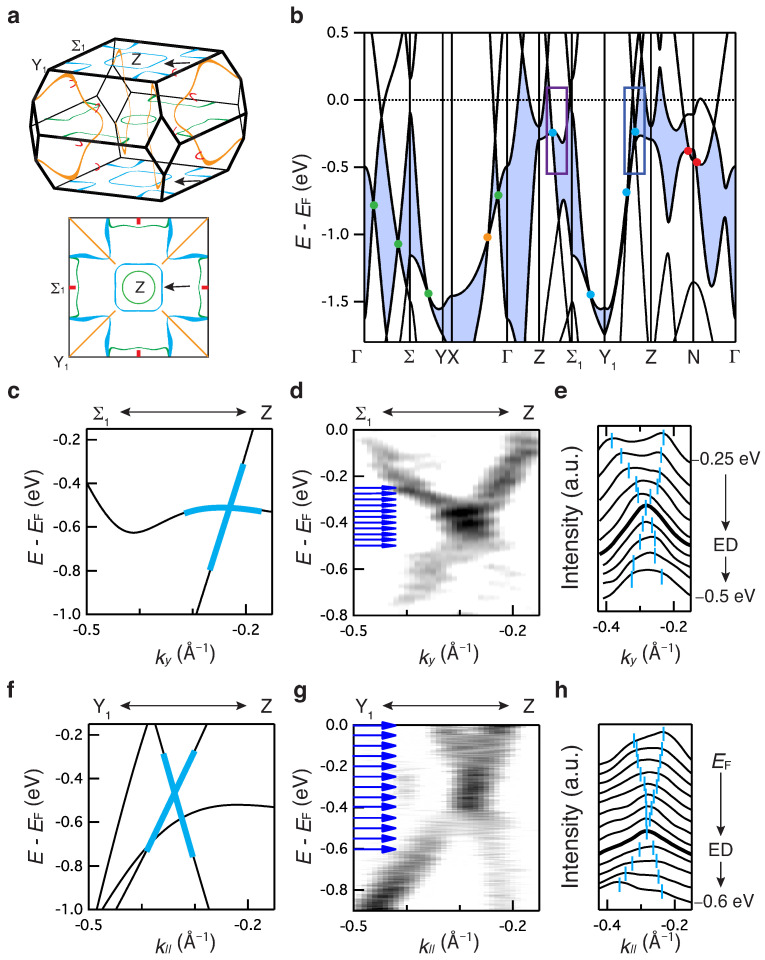
Bulk electronic structure of topological semimetal BaAl${}_{4}$. (**a**,**b**) The bulk Dirac nodal lines in bulk BZ (top in (**a**)) and on the (001) surface-projected BZ (bottom in (**a**)) and the calculated bulk electronic structure near $E_{F}$ without spin–orbit coupling (SOC) (**b**). The black arrows in (**a**) mark the experimentally identified nodal line in this work. The blue shaded region in (**b**) is the energy gap between the highest valence band and the second-highest valence band, where the nodal lines shown in this work exist. The blue and purple squares highlight the experimentally observed nodal line in this work. Each color of nodal lines/points in panels (**a**,**b**) represents the corresponding mirror plane shown in Figure 1b. (**c**,**f**) Zoom in of the calculated electronic structure along $\Sigma_{1}-$Z (**c**) and $Y_{1}-$Z (**f**) direction in $k_{z}=2\pi /c$ plane protected by $m_{100}$ (see the purple and blue squares in (**b**)). The solid light blue lines in the calculation are guides to the eye for the Dirac band-crossing. (**d**,**g**) The experimental electronic structure (second-derivative in momentum direction) along $\Sigma_{1}-$Z (**d**) and $Y_{1}-$Z (**g**) direction, respectively. (**e**,**h**) The momentum distribution curves (MDCs) along the cuts along $\Sigma_{1}-$Z (see blue arrows from −0.25 eV to −0.5 eV in (**d**,**e**)) and the cuts along $Y_{1}-$Z (see blue arrows from $E_{F}$ to −0.6 eV in (**g**,**h**)), respectively, showing the Dirac dispersion of the nodal line. The light blue marks indicate the peak positions. The bold black lines highlight MDC near the energy of Dirac point (ED).

Figure 2 shows the bulk electronic structure of BaAl${}_{4}$. Figure 2a shows the theoretical bulk nodal lines without SOC. The energy and momentum dispersions along the high symmetry directions reveal the presence of several nodal points within the valence bands for each mirror plane (see dots in Figure 2b). These nodal points, developing between the highest valence band and the second-highest valence band (Valence Gap (VG)), give rise to a variety of nodal lines in each mirror plane (see colored lines in Figure 2a). The colors used in Figure 2a,b for the nodal points/lines correspond to the same color scale used to represent the respective mirror plane (Figure 1b). A detailed analysis of the irreducible representations for each crossing is shown in Figure S1 in Supplementary Materials. Figure 2c,d show the calculated and experimental momentum and energy dispersions along the $\Sigma_{1}-$Z direction in the $k_{z}=2\pi /c$ plane. Following the maximum intensity, a Dirac-like linear-shaped dispersion can be observed, in agreement with the theoretical calculation in Figure 2c. The dispersion can be better extracted by following the peak positions in the momentum distribution curves (MDCs) shown in Figure 2e, where two peaks disperse linearly throughout the entire energy range and cross at ∼−0.4 eV, namely the Dirac point. The non-gap linear feature is further confirmed in Figure S5c in Supplementary Materials, where only one side of Dirac dispersion is observed due to the matrix element effect [42,43]. Figure 2f–h show the same as Figure 2c–e, respectively, but the momentum direction is the $Y_{1}-$Z direction in the $k_{z}=2\pi /c$ plane. Similar to the $\Sigma_{1}-$Z direction, the Dirac-like linear dispersion can also be observed in the $Y_{1}-$Z direction and is confirmed by the MDCs spectra, where the two peaks disperse linearly and cross at the ED. These Dirac nodes belong to one of the nodal loops in the $k_{z}=2\pi /c$ plane (see the black arrows in Figure 2a). The lack of a gap in the spectra can be due to the absence of hybridization between the two spins and/or weak SOC as in this case, where the bands near $E_{F}$ in the VG region are mainly composed of Al *s* and *p* orbitals (see details in Figure S2 of Supplementary Materials). Once SOC is introduced, the two spins are coupled and allowed to hybridize, resulting in a gap opening at each of the Dirac points/lines. This is true for each high symmetry direction, unless the Γ−Z direction, where the two bands belong to a different representation of the symmetry group, and therefore, their intersection is protected by the crystalline symmetry, $C_{4v}$. The details of the crystal symmetric information and topological nature with SOC can be found in ref. [30]. In the presence of weak SOC, the gap size becomes negligible, and this may be the case for the VG.

#### 3.1.1. Surface States

**Figure 3:**
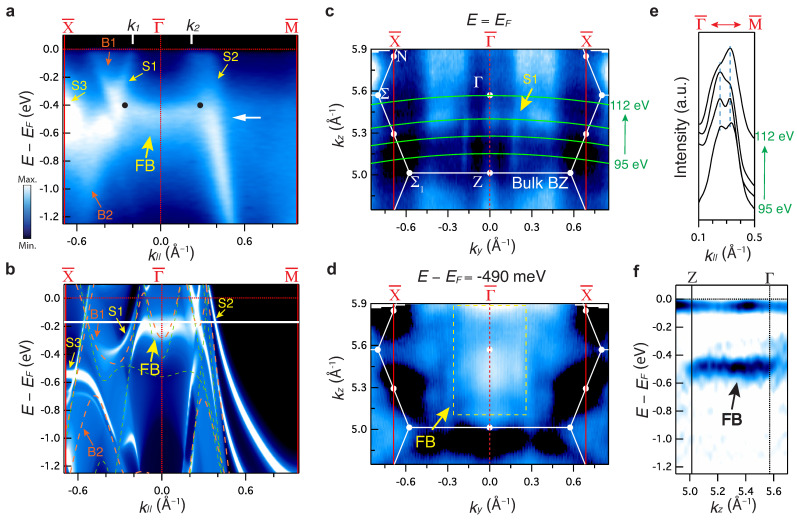
Surface states of BaAl${}_{4}$. (**a**) ARPES spectra of energy versus momentum cuts along the high-symmetry directions $\bar{X}-\bar{\Gamma }-\bar{M}$ with photon energies 95 eV. The orange and yellow arrows mark the observed bulk (B1–B2) and surface (S1–S3 and FB) states, respectively. The black dots represent the bulk Dirac nodes obtained from the bulk experimental results (Figure 2). (**b**) Calculated surface band structure along high-symmetry direction $\bar{X}-\bar{\Gamma }-\bar{M}$ of BaAl${}_{4}$ (001) surface for a semi-infinite slab. The orange dashed lines represent calculated bulk band structures without SOC for $k_{z}$ corresponding to the photon energy of 95 eV. The light-green dashed lines are calculated bulk for $k_{z}=2\pi /c$. (**c**) ARPES spectral intensity map in the $k_{z}-k_{y}$ plane at the $E=E_{F}$. The $k_{z}$ range covers half of the bulk BZ and corresponds to a photon energy range of 95–112 eV. The green solid lines represent the location of the cuts studied in this work. From bottom to top, each line corresponds to 95, 100, 105, and 112 eV of photon energy, respectively. (**d**) ARPES spectral intensity map in the $k_{z}-k_{y}$ plane at the binding energy E=−490 meV shown by the white arrow in panel (**a**). The yellow dashed rectangle represents the FB state. (**e**) MDCs of spectra along $\bar{\Gamma }-\bar{M}$ direction at $E=E_{F}$ for different photon energies represented in panel (**c**). The blue dashed lines represent the estimated peak positions. (**f**) Second derivative of energy versus $k_{z}$ intensity plot.

We now turn our attention to the surface-localized electronic states. Thanks to the matrix element effect, the bulk and surface electronic structures can be characterized selectively (see the detail in Figure S4 in Supplementary Materials). Figure 3 shows the surface electronic structure within the surface BZ projected onto the (001) plane. In addition to the bulk states identified in Figure 3b (labeled as B1–B2), three new sets of features are observed in Figure 3a: sharp, linearly dispersive states (labeled as S1–S2), a hole-like dispersion state (labeled as S3), and a weakly dispersive state (labeled as flat band (FB) state). The FB state connects the two Dirac nodes (see the black dots in Figure 3a) observed in Figure 2, indicating the direct relation with the observed nodal line. Similar features are observed in the surface state calculations shown in Figure 3b, pointing to their surface origin (see also Method section and Figure S3 in Supplementary Materials for more details about the calculation). These multiple surface states, originating from the Dirac nodal lines, imply that they are either topologically protected states or floating bands as a result of reduced symmetry at the surface [44,45]. Note that the surface state calculation is sensitive to the details of the simulations, as reported [46], and surface effects, such as potential band bending and structural relaxation effects, are not included in the calculation. These effects might lead to an apparent discrepancy between the experimental data (Figure 3a) and theory (Figure 3b). Other potential discrepancies could arise from matrix element effects [42,43]. These effects can, however, be minimized by changing the photon energy (see Figure S6 in Supplementary Materials), revealing different features. The surface origin of these states is further supported by their photon energy dependence (i.e., $k_{z}$ dependence) as shown in Figure 3c–f. Throughout the whole range, negligible dispersions are observed for each of these states, confirming their surface state origin. Indeed, S1 and S2 states form straight vertical lines, as indicated by the yellow arrows in Figure 3c and blue dashed line in Figure 3e, indicative of a lack of $k_{z}$ dispersion. On the other hand, the FB state defines a sheet in the ($k_{y}$, $k_{z}$) plane (see dashed yellow rectangle in Figure 3d), indicative of a localized state in $k_{z}$. This can be directly seen in Figure 3f, where the energy versus $k_{z}$ dispersion along the Z−Γ direction shows a localized two-dimensional state.

#### 3.1.2. Flat Surface Band

**Figure 4:**
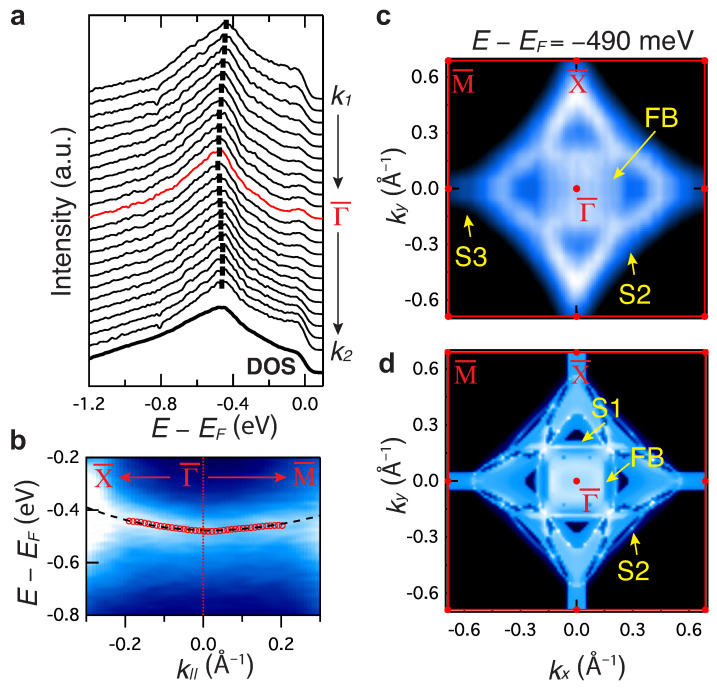
Full mapping of the topological drumhead surface state. (**a**) EDCs from $k_{1}$ to $k_{2}$ at the position indicated by the marks in Figure 3a. The bold line at the bottom is the integrated density of state (DOS). (**b**) Extracted dispersions of the drumhead state along $\bar{X}-\bar{\Gamma }-\bar{M}$ (the red dots) and the fitting (the black dashed line). (**c**) Constant energy maps at E=−0.49 eV. The surface states (S2–S3) and the flat band (FB) state are marked by the yellow arrows. (**d**) Calculated energy contour at the energy marked by white solid line in Figure 3c.

Among all the surface states, of particular interest is the FB state, which is the one appearing at E=−0.49 eV. Indeed, this state emerges out and connects the two Dirac bulk nodal lines, as expected in the case of a drumhead surface state (see also Figure 3a). Figure 4 presents its full momentum and energy characterization. The energy versus momentum dispersion (Figure 4b), extracted from the peak position of the energy distribution curves (EDC) (Figure 4a), appears weakly dispersing along both the $\bar{\Gamma }-\bar{X}$ and $\bar{\Gamma }-\bar{M}$ direction, with an overall bandwidth less than 38 meV and 23 meV, respectively, in the momentum range ($k_{1}$–$k_{2}$). This gives rise to an almost flat surface state with an effective mass of $m^{*}≃4.0m_{e}$ and $6.1m_{e}$ and a peak in the density of state at the energy of the FB state (see the bold line in Figure 4a). In Figure 4c, we show the momentum extension of the FB state. The constant energy map ($k_{y}$ versus $k_{x}$) at FB state shows that the FB state is localized in momentum along a well-defined filled square-shaped region, centered at the BZ center. The topology of the FB state is consistent with the confirmed bulk nodal loop (see Figure 2a) and is confined within an area of ∼0.16 Å${}^{-2}$. Additionally, the momentum location of the surface states S2–S3 is also visible in this energy window. In contrast, the main contribution of the S1 state appears near $E_{F}$ and is localized along a square-shaped region (see Figure S6 in Supplementary Materials). The topology of these states is qualitatively consistent with the theoretical constant energy map shown in Figure 4d. All the data reported so far support the existence of a strongly localized drumhead surface state in BaAl${}_{4}$ and reveal its extended location in momentum space.

## 4. Discussion

The localized nature of this state makes it unique with respect to previous studies [9,45,47,48], where highly dispersive FB states have been reported. Indeed, the flatness of our FB state drives a large density of states, as shown in Figure 3 and Figure 4, enhancing interaction effects significantly. The large area of the FB state is also promising in view of the enhancing of the electrons’ interaction [14,29], although the first step would be to engineer the FB state close to the Fermi level. Finally, it is noteworthy to point out that the observed bulk nodal line might be also responsible for the reported transport anomaly, including the quantum oscillation and extremely large magnetoresistance [30]. A complete quantification of the gap size in BaAl${}_{4}$, and hence the topological classification of BaAl${}_{4}$, requires more detailed calculations and further measurements. Even a small amount of lattice strain can tune the gap size [49,50,51,52,53], resulting in a unique Dirac semimetal, where both the Dirac point and Dirac nodal lines may coexist.

## 5. Conclusions

In summary, by combining surface-sensitive ARPES experiments with theoretical calculations, we have provided direct evidence for the existence of a flat extended surface state in a topological semimetal, BaAl${}_{4}$. We present that the flat surface state fills inside of the bulk square-shaped nodal line by showing the full momentum space topology of such a state. While further investigation is needed to conclude its topological origin, all the data reported here support that the observed flat band is directly associated with the observed Dirac nodal line and, therefore, the topological drumhead surface states. These results enable the exploration of such states to realize a number of novel correlated phases of matter [13,14,15,16,17,18,20,24,25,26,29,54].

## Data Availability

The data that support the finding of this study are available from the corresponding author upon request.

## Supplementary Materials

The following supporting information can be downloaded at: https://www.mdpi.com/article/10.3390/ma15082744/s1, A PDF file, including Figure S1: Irreducible representations for each crossing band in BaAl${}_{4}$; Figure S2: Calculated electronic structures with orbital weight; Figure S3: Wannier function for tight binding; Figure S4: ARPES spectral intensity map in the $k_{z}-k_{y}$ plane at the $E=E_{F}$ for wide momentum range; Figure S5: ARPES spectra of the bulk Dirac electronic structure; Figure S6: ARPES spectra with different photon energies and corresponding calculated bulk bands. Reference [30] is cited in the Supplementary Materials.
