# Supplementary material for: Observation of a Flat and Extended Surface State in a Topological Semimetal
Source: Materials (Basel). 2022 Apr 8;15(8):2744. doi: 10.3390/ma15082744 (PMC9026440; doi:10.3390/ma15082744)
Supplement: Supplementary file 1 [file materials-15-02744-s001.zip › materials-1666368-supplementary.pdf]

Supplementary Materials for Observation of a flat and extended surface state in a topological semimetal

Ryo Mori,[1,2] Kefeng Wang,[3,*] Takahiro Morimoto,[4,5] Samuel Ciocys,[1,6] Jonathan D. Denlinger,[7] Johnpierre Paglione,[3] and Alessandra Lanzara[1,6,†]

[1]*Materials Sciences Division, Lawrence Berkeley National Laboratory, Berkeley, California 94720, USA*

[2]*Applied Science & Technology, University of California, Berkeley, California 94720, USA*

[3]*Maryland Quantum Materials Center, Department of Physics, University of Maryland, College Park, Maryland 20742, USA*

[4]*Department of Applied Physics, The University of Tokyo, Hongo, Tokyo, 113-8656, Japan*

[5]*JST, PRESTO, Kawaguchi, Saitama, 332-0012, Japan*

[6]*Department of Physics, University of California, Berkeley, California 94720, USA*

[7]*Advanced Light Source, Lawrence Berkeley National Laboratory, Berkeley, California 94720, USA*

* Current address: Department of Physics and Astronomy, Rutgers, The State University of New Jersey, New Brunswick, New Jersey 08854, USA

† To whom correspondence should be addressed. Email: Alanzara@lbl.gov

Supplementary Note 1: Irreducible representations of crossing points

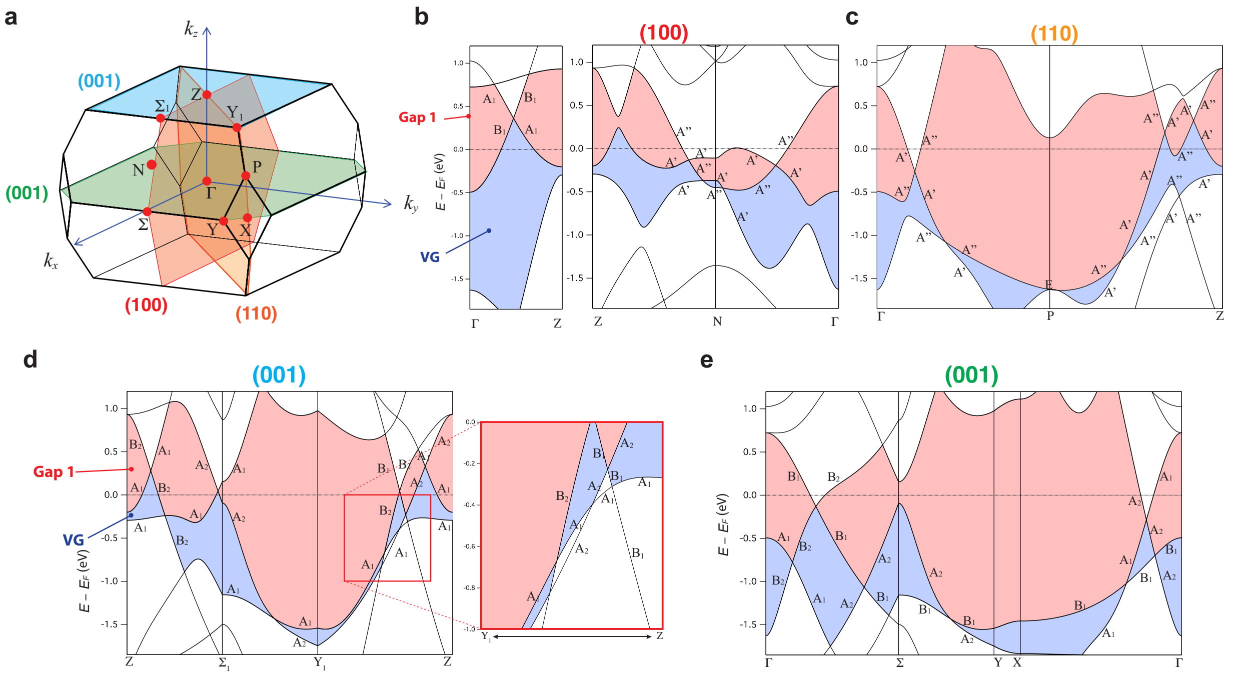

Figure S1. **Irreducible representations for each crossing band in $BaAl_4$. a** The bulk Brillouin zone (BZ) and the (001) surface BZ, marked with high-symmetry points. In the bulk structure, the three non-equivalent mirror-reflection planes m_{001} (green plane ($k_z = 0$) and light-blue plane ($k_z = 2\pi/c$)), m_{110} (orange plane), and m_{100} (red plane) are illustrated. **b** The calculated electronic structures with irreducible representations along high symmetry lines on the m_{100} plane; $\Gamma - \mathrm{Z}$ line (left panel) and $\mathrm{Z} - \mathrm{N} - \Gamma$ (right panel). The red shaded region (Gap 1) represents the energy gap between the lowest conduction and highest valence bands and the blue shaded region (Valence Gap (VG)) is the energy gap between the highest valence band and the second-highest valence band. **c** The calculated electronic structures with irreducible representations along high symmetry lines on the m_{110} plane. **d,e** The calculated electronic structures with irreducible representations along high symmetry lines on the m_{001} plane at $k_z = 2\pi/c$ (**d**) and $k_z = 2\pi/c$ (**e**)(left panels). Zoom-in of the electronic structure for $\mathrm{Y} - \mathrm{Z}$ (red square in the left panel in **d**) is shown in the right panel in **d**. The band crossings on the m_{100} plane, the m_{110} plane, and the m_{001} plane create the band closing line in both Gap 1 (right top panel) and VG (right bottom panel) as represented in Fig. 2(**b,c**) in the main text.

Figure S1 shows the irreducible representations of the band structures for each crossing point. The crystal structure of $BaAl_4$ has three non-equivalent mirror-reflection planes m_{001} (green and light-blue planes), m_{110} (orange plane), and m_{100} (red plane). The m_{110} plane and m_{100} plane have equivalent mirror planes along the orthogonal directions. The crossing bands on these mirror planes cannot hybridize because of the different irreducible representations of the space group where the two crossing bands belong to opposite mirror eigenvalues. Therefore, these crossing bands form nodal loops/lines on mirror planes.

Supplementary Note 2: Orbital contribution near E_F

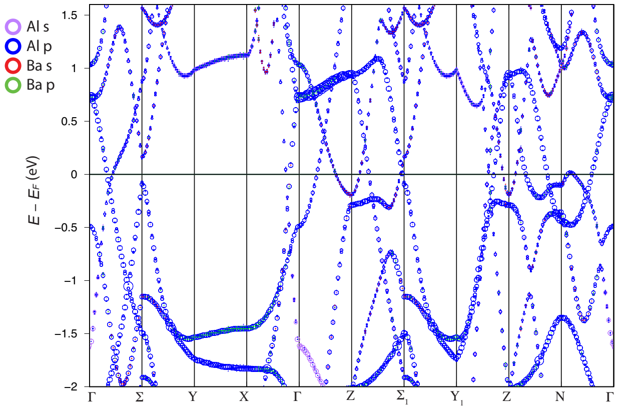

Figure S2. **Calculated electronic structures with orbital weight.** DFT calculated band structure with orbital weight. The size of the dots is proportional to the relative amplitude of the weight of orbital projection onto Al-s (purple), Al-p (blue), Ba-s (red), and Ba-p (green) orbitals. Ba s and p orbitals are represented by red and green. The Fermi level is set to zero.

To see the orbital contribution for the band structures near E_{F}, the orbital projected band dispersions are calculated as shown in Figure S2. The size of circles represents the relative weight of orbital projection onto Al-p, Al-s, Ba-s, and Ba-p orbitals. The bands near E_{F} are composed mainly of Al-p and Al-s orbital, resulting in small SOC effects. Once SOC is included in the calculation, hybridization leads to gap opening. The size of the gap is directly related to the strength of SOC.

Supplementary Note 3: Wannier function for tight-binding

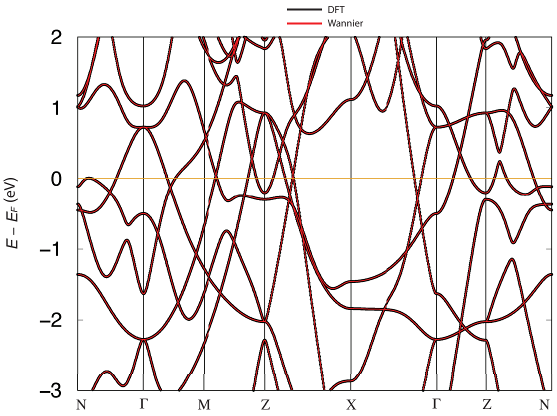

Figure S3. **Comparison Wannier function based tight-binding model with DFT.** Band structure from Wannier function based tight-binding model (red) and DFT (black). Wannier effective tight-binding model reproduces all the features calculated with DFT.

To help understand the observed electronic structures in the main text, we create Wannier function based tight-binding model. Figure S3 shows the band dispersions based on DFT (black lines) and Wannier functions (red lines). This tight-binding model can well reproduce the energy bands near E_F, which are crucial to the band topology. This Wannier function-based model is used in the surface electronic structure calculation shown in the main text.

Supplementary Note 4: Bulk and surface electronic structures

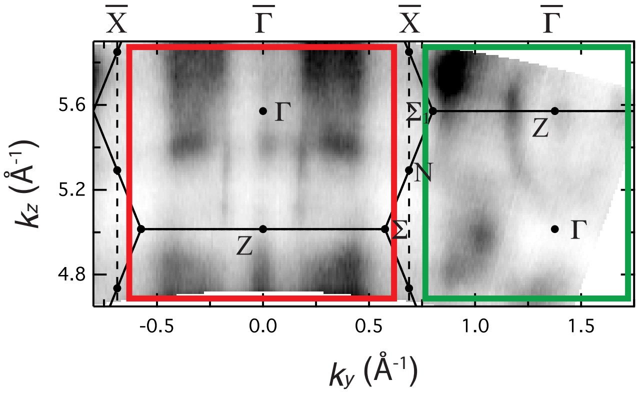

Figure S4. **ARPES spectral intensity map in the $k_z - k_y$ plane at the $E = E_\mathbf{F}$ for wide momentum range.** The black solid (dashed) lines represent the bulk (surface) BZ. The red and green square mark the surface and bulk sensitive region in BZ, respectively.

Thanks to the matrix element effect, the bulk and surface electronic structures can be characterized selectively. In Figure S4, we show the out-of-plane Fermi surface mapping taken with the different photon energies. In the red square region in the panel, negligible dispersions are observed throughout the whole range, confirming their surface state origin. On the contrary, the dispersive electronic structures are observed in the different BZ (see the green square region), suggesting their bulk state origin. The observed bulk states here are consistent well with the calculated Fermi surface (see Ref [30] for more details about the bulk structure).

Supplementary Note 5: Bulk Dirac nodes

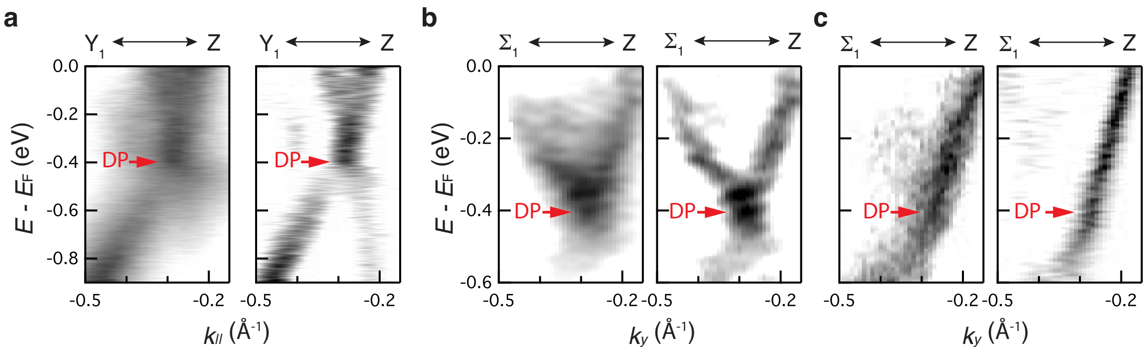

Figure S 5. **ARPES spectra of the bulk Dirac electronic structure. a** ARPES spectra (left) and its second derivative in momentum (right) along Y_1-Z direction. **b** ARPES spectra (left) and its second derivative in momentum (right) along Σ_1-Z direction. **c** ARPES spectra (left) and its second derivative in momentum (right) along Σ_1-Z direction, taken in the different BZ from **b**. The red arrows show the Dirac point (DP).

As shown in Figure 2 in the main text, the bulk Dirac nodes along two high-symmetric lines in $k_z = 2\pi/c$ plane are observed. Figure S5(a)-(b) show the raw normalized spectra and their second derivative along Y_1-Z direction. and Σ_1-Z, respectively. Panel (c) shows the experimental result along Σ_1-Z direction (the same direction as panel (b)), but taken in the different BZ. In this BZ, the only one side of Dirac dispersion is observed due to the matrix element effect, further confirming the non-gap linear dispersion.

Supplementary Note 6: Photon energy dependence of bulk and surface states

Photon energy dependence is a powerful way to extract the k_z dependence of electronic structures. The k_z dependence can be obtained by changing photon energy using the free-electron final state approximation:

$$k_z = \frac{1}{\hbar}\sqrt{2m_e(E_i + h\nu - \Phi)\cos\theta + V_0} \tag{1}$$

where m_e is the free electron mass, E_i is the energy of the initial state, $h\nu$ is the photon energy, Φ is the work function, θ is the emission angle of photoelectrons, and V_0 is the inner potential. Electronic structures taken with the different photon energies resolve the surface nature of the observed spectra; surface states are two-dimensional states and hence showing no k_z dependence, while bulk states show k_z dispersive three-dimensional feature.

Figure S6 shows the experimental spectra (panels (a–h)) and the calculated bulk structures for several different photon energies, corresponding to different k_z values in panels (i–l); $k_z \sim 0$ ($h\nu = 112$ eV in (**i**)), $k_z \sim 0.6\pi/c$ ($h\nu = 105$ eV in (**j**)), $k_z \sim 1.05\pi/c$ ($h\nu = 100$ eV in (**k**)), and $k_z \sim 1.5\pi/c$ ($h\nu = 95$ eV in (**l**)). The Fermi surface mappings (panels (a)-(d)) confirm that the topology of the observed surface states (S1-S2) near E_F is consistent with the theoretical constant energy map shown in Figure 4(d) in the main text. By comparing the experimental spectra (panels (e)-(h)) and the bulk calculations (panels (i)-(l)), the

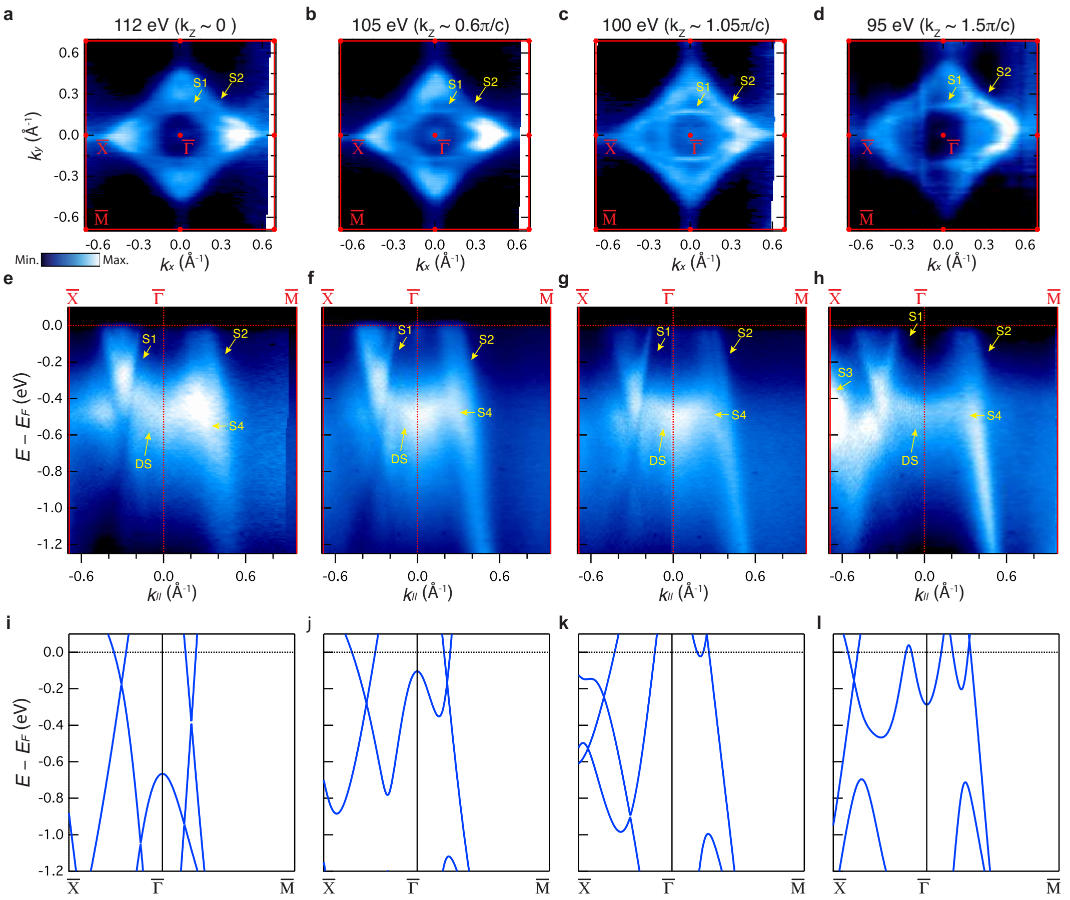

Figure S6. **ARPES spectra with different photon energies and corresponding calcu-lated bulk bands. a–d** Fermi surface mappings with different photon energies 112 (**a**), 105 (**b**), 100 (**c**), and 95 (**d**) eV. **e–h** ARPES spectra of energy versus momentum cuts along the high-symmetry directions with photon energies 112 (**a**), 105 (**b**), 100 (**c**), and 95 (**d**) eV. **i–l** Calculated bulk band structures without SOC. Each structure is calculated for k_z corresponding to each pho-ton energy; $k_z \sim 0$ ($h\nu = 112$ eV in (**i**)), $k_z \sim -0.6\pi/c$ ($h\nu = 105$ eV in (**j**)), $k_z \sim -1.05\pi/c$ ($h\nu = 100$ eV in (**k**)), and $k_z \sim -1.5\pi/c$ ($h\nu = 95$ eV in (**l**)).

S1–S4 states and the DS state are clearly distinguished from the bulk electronic features, validating their surface origins. Note that the matrix element effects play a different role for different photon energy, leading to the different appearance of these states. Indeed, S4 appears in panels (e)-(g) and the intensity is suppressed in panel (h), and S3 appears in panel (h).
